# How society treats smoking

**DOI:** 10.1186/2045-4015-1-29

**Published:** 2012-07-24

**Authors:** Gregory N Connolly

**Affiliations:** 1 Harvard School of Public Health, Landmark Building, 401 Park Drive, 3rd Floor, Boston, MA 02115, USA

**Keywords:** Smoking, Social change, Translational research

## Abstract

Bans on smoking in bars and other public places can make an important contribution to public health. However, for these bans to be effective, they require broad public support. Qualitative studies of the attitudes and perceptions of bar owners and patrons can help public health professionals identify the steps needed to promote public support for smoking bans. Such studies can also generate narratives and quotes that can help public health professionals translate findings on perceptions and attitudes into effective public education campaigns and related policy changes.

## 

In this month’s *IJHPR*, Orna Baron-Epel and colleagues explore some of the unique challenges that the State of Israel faces in translating scientific findings on the harmful effects of second hand smoke into improved health for nonsmokers. They do so through a qualitative study of compliance with a law banning smoking in bars and pubs [1]. This is not the first, nor will it be the last, article on this complex public health practice that entails competing interests between sectors of society – in this case, public health and bar owners. As will be discussed further below, the study underscores the need for a sophisticated and coherent approach to knowledge translation, which includes both quantitative measures of disease risk as well as qualitative measures of attitudes, which together contribute to social cohesion for better health.

## The evolution of societal attitudes toward smoking in public places

Many societies have evolved from a history of rewarding smokers with social esteem to using public health policy to combat smoking through increasing social isolation, thereby denying the individual the nurturing effects of relationships with others [2]. This evolution has been driven by the creation of new science showing harm, including lung cancer in healthy exposed nonsmokers, and highlighting the need for protection by public health policies [3]. In some countries, these findings have been incorporated into broader views that rights and justice side more with the nonsmokers who are harmed and unable to protect themselves, than with the smokers or bar owners who believe that prohibiting the smoking of toxin-rich tobacco is an affront to individual autonomy from overzealous health advocates [2].

Society’s treatment of smoking is complex and has radically changed over the years in the United States and other western nations. The great American actor Humphrey Bogart ("Bogey") smoked cigarettes in the classic movie *Casablanca* and earned an Oscar Award, while today smokers are forced to stand isolated outside of theaters.

Accordingly, it is surprising to see this issue still being debated today in Israel, given Israel's relatively low smoking rate of 20% (which is lower than that of supposedly "smoke-free" America), its high education level, the nation’s excellent health care system, some of the world's best public health prevention programs, and an enduring civic obligation for government to protect and maximize well-being. In this context, it is particularly perplexing and troubling that many bar owners and patrons in Tel-Aviv and other Israeli cities are allowed to simply ignore the law banning smoking in public places.

As such, a study of the causes of this troubling situation may generate insights that can be beneficial to the dozens of other nations faced with similar situations in their hospitality industries, but with far less social cohesion or resources than Israel has, to make the recently enacted Clean Air laws work. Of even larger interest is the charting of a new path for creating knowledge applicable to the increasingly complex world of public health decision making in the 21st century that complements studies that quantify disease risk with a better understanding of how they can be translated into practice.

## The vital role of social factors in promoting clean air policies

According to research in California and Massachusetts, the social treatment of smoking is far more effective than individualized patient interventions [4]. Of course, the greatest effects can be achieved when social and individual interventions go hand in hand (i.e., when societies both change the social norms and provide direct assistance to individuals seeking to quit smoking). Yet, without the social interventions, little can be expected from medications or personalized counseling when a non-compliant bar fosters relapse. Using medications or cognition to fight a larger group behavior may not be effective in bringing about large-scale changes. Changing the social norms certainly is [5].

Why is this the case? A major factor appears to be that humans thrive, heal, and create new knowledge through social interaction. Those relegated to outside areas that look like polluted, overpopulated fish bowls may find the need to be with others (which formerly functioned as a social cue to smoke) so strong that the cravings and urges to smoke are extinguished, and the urge to be with others fosters a resolve to quit.

The study by Baron-Epel and colleagues shows how the bar owners’ misperception of the economic effect of smoking bans limits the enforcement of Israel's law on smoking in public places. The study suggests that better enforcement could make an important contribution to Israeli society, and the study may even provide some important insights for other nations as well. Hopefully, if it succeeds in helping Israel do a better job of enforcing its own clean air laws, Israel's citizens will be better protected, more Israeli smokers will quit, and the tobacco industry (which is based overwhelmingly outside of Israel) will be denied new opportunities to pass the smoking epidemic down to the next generation of Israelis in the smoke-filled nicotine classrooms of Israeli pubs and bars. The study may also give us insights into the types of knowledge needed for public health decision making in this century.

## Cross-national differences in the adoption and implementation of clean air policies

The vast majority of nations including Israel have ratified the World Health Organization’s Framework Convention on Tobacco Control (FCTC), one of whose cornerstones is clean air policies [6]. Not surprisingly, the United States Senate has not ratified the FCTC. The Washington-based tobacco interests possibly see it as a global roadblock to further expanding U.S. hegemony of its control of the world tobacco trade. Yet, the adoption of the FCTC in most countries around the world has created an environment of change even in America, where local citizens and local governments (rather the central government) have become the driving force for clean air laws [7]. The first municipal clean air law was adopted in San Francisco over thirty-five years ago and then spread to many U.S. cities regardless of social status. This story of grass roots success remains unparalleled in U.S. public health history.

Subsequent to the adoption of FCTC by the WHO in 2004, many nations quickly passed clean air laws, only to find that the intent of the law was not realized due to lack of enforcement and insufficient investment in public education on the dangers of secondhand smoke. For example, in 1989 the French Parliament passed a total ban on smoking in all bistros, but failed to tell the public what it had done. As a result, "smoky” bistros remained the norm. More recently, France “reenacted” the law, but this time enactment was preceded by a distinctively French advertising campaign that stressed the universal protection and the esprit of a Smoke-free society. Viola! This time round, the ban worked [8].

Across the channel, the Irish banned smoking in all pubs, only to build enclosed suites for smokers just outside the pubs, some of which even included heaters and televisions for the ostracized. As a result, despite the ban, the smoking rate in Ireland has been stuck at around 28% and the expected decline in cigarette consumption never materialized [9]. A new social practice quickly emerged called “smirting”, where young Dubliners smoke and flirt with members of the opposite sex, and with cigarettes quickly regaining the sexual attraction long associated with a lip-hanging Luck Strike. Like the 1989 French experience, no education campaign was carried out in Ireland, abandoning the media to tabloid stories of opposition. This, in turn, undermined the social cohesion needed to enforce a ban on smoking in public places.

Many nations still disdain bans on smoking in public areas as an infringement on personal rights. Yet, this view, which is fueled in the U.S. among bar and pub owners by industry support, should be tempered with the understanding that autonomy and individual rights do not justify harm to others and that clean air is a public good, leading to better health for all. The realization that bars have become nicotine classrooms for the next generation of smokers is a cause for concern. The club setting has strong cues that associate smoking with pleasure and also cue relapse based on a learned behavior [10]. This is further "juiced" by a few drinks that dull the pointers on avoidance that were included in a quit plan drawn up years before.

## The crucial role of grass roots support for clean air policies in the U.S. and beyond

The experience of successful adopters may clarify these findings. Clean Indoor Air laws were adopted laboriously over decades in the U.S. where the developing policy was based on knowledge of the injustice and moral failure to protect the vulnerable who were exposed to harm. The tobacco industry’s aggressive opposition only confirmed that economics, and not people, were central to the problem.

This sense of justice and moral virtue was espoused by the founders of America, as well as the founders of Israel, but it has subsequently become increasingly lost in government. Despite this, governments thrive on the actions of the people. Creation of cohesion for a public health law must be based on solid science to support the social will. This was achieved in the U.S. through reports of the Surgeon General [3] and the Environmental Protection Agency [11] that summarized the known science and concluded that the only measure that provides protection is a comprehensive ban.

These national reports had little direct effect in Washington. For example, measures to curb smoking in the workplace nationwide that were considered by the Occupational Health and Safety Commission during the Clinton presidency were turned down. However, the reports did lead to a ban on smoking on airplanes. They also empowered common citizens (both in the U.S. and overseas) with the tools to discuss with local authorities the problems as well as possible solutions. Scientific studies showing no economic harm to the hospitality industry in communities that adopted comprehensive policies were published and then used to refute false economic accusations [12]. However, the central focus of the scientific effort was the health benefits of smoking bans, particularly for non-smoking workers and children, with the obvious implications for moral justice.

Attempts by the industry to create doubt and weaken the growing cohesion were met by public opinion polls and other surveys showing widespread support for clean air legislation and that the only entity to suffer from a clean air policy was the tobacco industry. As in Israel, 80% of U.S. citizens don’t smoke and if they could dine or drink in a healthy environment their overall increase in their patronage would far outweigh any loss of patronage from smokers.

A popular acronym was coined for the process – “KILLS”, standing for "Keep It Local and Loud, Stupid". Public education campaigns on the dangers of smoking provided air cover to the ground troops enforcing the laws, and further created the cohesion needed for successful implementation. Within the Commonwealth of Massachusetts, complementing the growing public knowledge was the political will of the Mayor of Boston who led 32 neighboring communities to adopt common policies simultaneously. California, another state with a large-scale tobacco control program, boasts that its treatment of tobacco dependence is not based on purchase of expensive medications for individual smokers but rather on having the smoker work and live in smoke-free environments [4].

Like California, the Commonwealth of Massachusetts treats smoking as a disease rather than as a tool to gain social esteem. In 1955, over half of Massachusetts physicians smoked. Today smoking is banned across the entire Harvard Medical area, including the lawns and gardens. Research with smokers is also banned at the Harvard Medical School, unless an explicit exemption has been granted by the State. Even if such research was allowed, subject recruitment would be prohibitive given the daily smoking rate of 12% [13].

Preventing the tobacco industry from using bars as classrooms for tobacco addiction in the young and providing cues for relapse among quitters should be a vital component of any nation's tobacco control efforts. However, society and its members must be "pre-vaccinated" with information about the importance of such a prohibition for the policy to work. Local discussion and debate can contribute greatly to dialogue among those of opposing views and meaningful debate, expert knowledge on harm, public education, and political leadership as the basis for greater enforcement. By 2004, when the then-reluctant Governor Romney signed into law a state-wide ban, over 85% of the state’s population was already covered by local laws [14]. Fears of wide spread violations were met by self-enforced compliance.

## The combined role of quantitative and qualitative in promoting clean air policies

To date, in an effort to use scientific findings to advance public health policy, quantitative researchers have conducted over fifty studies across the globe (including in Israel) measuring respirable small particles (RSPs) associated with second hand smoke in the air of indoor places [15-19]. They do so using small, high tech devices to quantify the level of exposure to toxins. The results are compared to established air quality standards with the hope that the scientific findings will be sufficient for passage of a law and assure compliance.

However, relying solely on the numbers and probability to advance public policy clearly has its shortcomings. Baron-Epel and colleagues have taken a complementary approach through qualitative research on the perceptions and attitudes of bar owners – a group with a clear interest and bias.

It may well be that public health policy can benefit greatly from a synthesis between quantitative measurement at the chemical level and qualitative studies of social forces that deter science translation. In the 21st century, public health practice is rapidly changing, as is the knowledge base used to advance it. In preceding centuries, public health benefited from unfettered authority to control contagious disease and ensure that the health market place was safe and effective. But, this singularity of authority may be a relic of a bygone era. Today, public health sits at a much larger table with shared decision makers, consisting of political, economic, legal, religious, or even tobacco industry stakeholders. They may share common concerns for far different reasons and have far different solutions than those that pure science warrants. In such forums, the public health knowledge broker of yesterday armed with quantified measures of risk may be seen more as a threat than a scientific source for solving the problem. The broker may be perceived as a foreigner in a world driven by marketplace economics, who doesn’t even speak its language. Given this brave new world, the experience with curbing exposure to the toxins of second hand smoke may require an expansion of knowledge sources and different communication tools that combine both qualitative and quantitative sources translated into stories of relationships among individuals and social groups that the stakeholder understands and that the knowledge broker can easily relate.

Another dimension of knowledge that is being quickly lost is not science but the knowledge of justice and moral virtue as they apply to the interrelationships between the individual and social group. Sandel, in his new book *What Money Can’t Buy*[20], argues that the science of market economics has entered virtually every phase of the human experience and by its nature cannot assign value to the intangible, such as the value of health. He advocates for setting moral limits on the marketplace but leaves the solution to the reader.

Baron-Epel and colleagues chose to study a group at the table (i.e., bar owners) whose main concerns are not RSPs or even worker health, but the number of shekels or patrons lost if a bar went smoke-free. But, it has been said that the truth often lies with one’s enemies, so the perspectives of bar owners and smoking patrons should not be ignored. Moreover, the qualitative nature of the study provides colorful, human interest stories that can then be brought to today's complex decision making table as part of an expanded knowledge base.

Interestingly, the article by Baron-Epel et al. generates many of the same policy conclusions that would be generated by an RSP study, with regard to the need for societies to do more to meet the challenges of poor compliance with a sound, socially-supported, public health law. The authors appropriately call for political leadership to enforce the law in a manner that is fair, consistent, and involves effective penalties. It is the public’s responsibility to create the necessary social cohesion through active learning, such as the simple placement of "no smoking signs" in pubs.

The unique contribution of hard science studies, such as the measurement of RSP levels in bars and pubs, is that they can provide objective, quantifiable measures of the extent of public health hazards, including how they differ across settings and change over time. The unique contribution of qualitative studies of the attitudes and perceptions of bar owners is that they can help public health professionals identify the steps needed to promote public support for smoking bans. Such studies can also generate stories and quotes that can help public health professionals translate findings on perceptions and attitudes into effective public education campaigns and related policy changes. Clearly, the synthesis between quantified measurement of physical conditions and qualitative explorations of attitudes and perceptions can contribute greatly to the advancement of population health.

Oddly, a study similar to this one was conducted in the late 1990s in California among bar owners and workers, where they painted a future of doom and gloom. When the study was repeated three years later the results were exactly opposite – the owners reported that business was booming in bars filled with clean air!

## 

Commentary on the paper by Baron-Epel, Satran, Cohen, Drach-Zehavy, Hovell MF: **Challenges for the Smoking ban in Israeli Pubs and Bars: Analysis Guided by the Behavioral Ecological Model.**

## Competing interests

The author declares that he has no competing interests.

## Author information

Gregory N Connolly is the Director of the Center for Global Tobacco Control at the Harvard School of Public Health, where he is also Professor of the Practice of Public Health in the Department of Society, Human Development and Health. His research interests lie in establishing a science base for tobacco product regulation. Professor Connolly advises health system leaders in many countries around the world, including Cyprus, Greece, Ireland, South East Asia, and Israel, on the development of effective tobacco control policies and the need for evaluations of their impact on population health. In 1990, he led U.S. efforts to end the use of trade sanctions to compel foreign nations to import U.S. cigarettes.
